# Polk’s Medical Register and Directory of North America

**Published:** 1904-05

**Authors:** 


### BOOK REVIEWS.

POLK'S MEDICAL REGISTER AND DIRECTORY OF NORTH AMERICA, Eighth Revised Edition 1904, has been issued. This is the standard and covers the field as no other work can. The present volume seems even more complete than its predecessor.

Institutions and physicians needing a cyclopedic medical directory will find it in Polk's.

